# Minieditorial: “Criação e Implementação de um Banco de Dados Prospectivo e Multicêntrico de Pacientes com Infarto Agudo do Miocárdio: RIAM”

**DOI:** 10.36660/abc.20200158

**Published:** 2020-04-06

**Authors:** José Mariani

**Affiliations:** 1 Hospital Israelita Albert EinsteinSão PauloSPBrasilHospital Israelita Albert Einstein,São Paulo, SP, Brasil; 2 Santa Casa de São PauloSão PauloSPBrasilSanta Casa de São Paulo,São Paulo, SP, Brasil; 3 Instituto do CoraçãoHCFMUSSão PauloSPBrasilInstituto do Coração – HCFMUSP,São Paulo, SP, Brasil

**Keywords:** Doenças Cardiovasculares/mortalidade, Epidemiologia, Infarto do Miocárdio/prevenção e controle, Política de Saúde Pública, Base de Dados, Tomada de Decisões

As doenças cardiovasculares, incluindo o infarto agudo do miocárdio (IAM), representam importante problema de saúde pública no Brasil e no mundo, com elevadas taxas de incidência e mortalidade.^1^ A taxa de mortalidade brasileira por esse grupo de causas (183,3/100.000)^1,2^ encontra-se entre as maiores do mundo e é semelhante à de países como a China e do Leste Europeu.^1,3^

A instituição de políticas de promoção de saúde, diagnóstico precoce e tratamento efetivo estão entre as mais importantes estratégias de prevenção e tratamento dessas doenças, que ainda correspondem à principal causa de mortalidade em todo o mundo em pacientes adultos. Porém, o reconhecimento bem como a mensuração real e efetiva dessas doenças ainda estão dentre as estratégias a serem otimizadas no nosso meio.

Assim, faz-se necessário conhecer e quantificar efetivamente os pacientes com diagnóstico de IAM para que medidas efetivas e assertivas possam ser tomadas na tentativa de reduzir sua morbimortalidade. Uma das maneiras mais conhecidas para esse tipo de mensuração consiste na elaboração de um banco de dados representativo e de fácil acesso e interpretação. Ainda assim, esbarramos em mais um problema, que consiste em como fazê-lo e implementá-lo. Boa parte dessas questões foram respondidas elegantemente no artigo elaborado por Vaz et al.,^4^ intitulado: “Criação e Implementação de um Banco de Dados Prospectivo e Multicêntrico de Paciente com Infarto Agudo do Miocárdio: RIAM”.^4^ A descrição clara e objetiva do passo a passo para a criação, implementação e expansão desse banco de dados constitui literatura rara e imperdível para todos os envolvidos na abordagem dessas patologias.

A possibilidade de termos acesso a informações geradas por bancos de dados bem desenhados e confiáveis oferece uma fotografia mais próxima da nossa realidade, aumentando a eficácia na tomada de decisões e tornando a gestão financeira mais eficiente, o que é crucial para nossas políticas de saúde pública.
