# Supplementary material for: Minieditorial: “Criação e Implementação de um Banco de Dados Prospectivo e Multicêntrico de Pacientes com Infarto Agudo do Miocárdio: RIAM”
Source: Arq Bras Cardiol. 2020 Apr 6;114(3):456. [Article in Portuguese] doi: 10.36660/abc.20200158 (PMC7792728; doi:10.36660/abc.20200158)
Supplement: Translation

# Translation: Short Editorial: “Creation and Implementation of a Prospective Multicenter Registry of Acute Myocardial Infarction: RIAM”

**Authors:** José Mariani

**Affiliations:** 1 BrazilHospital Israelita Albert Einstein, São Paulo, SP - Brazil; 2 BrazilSanta Casa de São Paulo, São Paulo, SP - Brazil; 3 BrazilInstituto do Coração – HCFMUSP, São Paulo, SP - Brazil

**Keywords:** Cardiovascular Diseases/mortality, Epidemiology, Myocardial Infarction/prevention and control, Public Health Policy, Database, Decision Making

Cardiovascular diseases, including acute myocardial infarction (AMI), represent a major public health issue in Brazil and worldwide, with elevated incidence and mortality rates.^1^ The rates of Brazilian mortality for this group of causes (183.3/100,000)^1,2^ are amongst the highest in the world and is similar to that of countries such as China and areas such as the east of Europe.^1,3^

The implementation of health promotion policies, early diagnosis and effective treatment are some of the most important prevention and treatment strategies for these diseases, which remain the leading cause of mortality among adult patients globally. However, the recognition of these diseases, as well as their real and effective measurements, are among the strategies that must be optimized in our field.

Thus, it is necessary to effectively identify and quantify patients with a diagnosis of AMI so that effective and assertive measures can be taken in an attempt to reduce their morbidity and mortality. One of the best-known measurement techniques is the creation of a representative database that can be easily accessed and interpreted. But here we run into yet another problem: how to create and implement it. Much of these questions were elegantly answered by Vaz J. et al.,^4^ in their article entitled: “Criação e Implementação de um Banco de Dados Prospectivo e Multicêntrico de Paciente com Infarto Agudo do Miocárdio: RIAM” (“Creation and Implementation of a Prospective Multicenter Registry of Acute Myocardial Infarction: RIAM”).^4^ The clear and objective description of the steps required to create, implement and expand these databases provides a unique and unmissable literature for all involved in the approach of these pathologies.

The possibility of having access to information generated by well-designed and reliable databases provides a framework closer to Brazilian reality, increasing decision-making efficacy and improving financial management, which is of paramount importance for public health policies.
